# Transcriptome profiling of fruit development and maturation in Chinese white pear (*Pyrus bretschneideri* Rehd)

**DOI:** 10.1186/1471-2164-14-823

**Published:** 2013-11-23

**Authors:** Min Xie, Ying Huang, Yanping Zhang, Xin Wang, Hua Yang, Oliver Yu, Wenhao Dai, Congbing Fang

**Affiliations:** School of Horticulture, Anhui Agricultural University, Hefei, 230036 Anhui P R China; Key Laboratory of Tea Biochemistry & Biotechnology, Ministry of Education, Anhui Agricultural University, Hefei, 230036 Anhui P R China; Department of Plant Sciences, North Dakota State University, Fargo, ND 58108 USA

## Abstract

**Background:**

Pear (*Pyrus spp*) is an important fruit species worldwide; however, its genetics and genomic information is limited. Combining the Solexa/Illumina RNA-seq high-throughput sequencing approach (RNA-seq) with Digital Gene Expression (DGE) analysis would be a powerful tool for transcriptomic study. This paper reports the transcriptome profiling analysis of Chinese white pear (*P. bretschneideri*) using RNA-seq and DGE to better understand the molecular mechanisms in fruit development and maturation of Chinese white pear.

**Results:**

*De novo* transcriptome assembly and gene expression analysis of Chinese white pear were performed in an unprecedented depth (5.47 gigabase pairs) using high-throughput Illumina RNA-seq combined with a tag-based Digital Gene Expression (DGE) system. Approximately, 60.77 million reads were sequenced, trimmed, and assembled into 90,227 unigenes. These unigenes comprised 17,619 contigs and 72,608 singletons with an average length of 508 bp and had an N_50_ of 635 bp. Sequence similarity analyses against six public databases (Uniprot, NR, and COGs at NCBI, Pfam, InterPro, and KEGG) found that 61,636 unigenes can be annotated with gene descriptions, conserved protein domains, or gene ontology terms. By BLASTing all 61,636 unigenes in KEGG, a total of 31,215 unigenes were annotated into 121 known metabolic or signaling pathways in which a few primary, intermediate, and secondary metabolic pathways are directly related to pear fruit quality. DGE libraries were constructed for each of the five fruit developmental stages. Variations in gene expression among all developmental stages of pear fruit were significantly different in a large amount of unigenes.

**Conclusion:**

Extensive transcriptome and DGE profiling data at five fruit developmental stages of Chinese white pear have been obtained from a deep sequencing, which provides comprehensive gene expression information at the transcriptional level. This could facilitate understanding of the molecular mechanisms in fruit development and maturation. Such a database can also be used as a public information platform for research on molecular biology and functional genomics in pear and other related species.

**Electronic supplementary material:**

The online version of this article (doi:10.1186/1471-2164-14-823) contains supplementary material, which is available to authorized users.

## Background

The genus *Pyrus* is one of the most important genera in Rosaceae family for fruit production. *Pyrus* species are widely used for commercial fruit production in 76 countries or regions around the world (http://faostat.fao.org) and their economic importance has been well recognized [1]. There are four major edible species in *Pyrus*: *P. communis* L. is mainly cultivated in Europe, North America, South America, Africa, and Australia. The other three species, *P. bretschneideri* Rehd., *P. ussuriensis* Maxim., and *P. pyrifolia* (Burm.) Nakai., are grown in East Asian [1, 2]. The world production of pear has doubled in the past 16 years. China is the largest pear producer. In 2010, China produced 15.23 million tons (Mt) of pear fruits that accounted of 67.26% of world pear production (22.64 Mt) (FAOSTAT, 2012).

As of July 2013, 26,696 nucleotide sequences, 4,413 expressed sequence tags (ESTs), 52 genome survey sequences (GSS), and 2,636 proteins from the *Pyrus* genus have been deposited in GenBank. These sequences mostly derived from cDNA cloning and EST sequencing [3–7] provide useful information for transcriptional analysis, candidate gene discovery, and gene functional analysis; however, a comprehensive description of genes that expressed in pear fruit during the fruit development and maturation period remains unavailable.

Recent years, use of RNA-seq, the next generation sequencing technology, has generated over one billion bp of high-quality DNA sequence per analysis/experiment [8] and has dramatically improved the efficiency of gene discovery and functional analysis [9, 10], which largely facilitates the investigation of the functional complexity of transcriptomes [11, 12]. Illumina sequencing of transcriptomes for yeast [13, 14], *Arabidopsis*[15], mouse [16, 17], and human cells [18, 19] has confirmed that next generation sequencing is well-suited for surveying transcriptome profiles in eukaryotes. Recently Illumina RNA-seq system has been used to identify genes related to bud dormancy in pear (*P. pyrifolia*) [20]. RNA-seq is not limited to detecting transcripts for organisms with existing genomic sequences; it also can be used to sequence non-model organisms that lack of genomic information [21–25]. DGE is a tag-based transcriptome sequencing approach in which the expression level of all genes in a sample is measured by counting the number of individual mRNA molecules produced from each gene, which enables the DGE protocol more suitable and affordable for comparative gene expression studies [25–31]. Despite its obvious potential, the next generation sequencing has not yet been applied to pear research.

In this study, 5.47 gigabase pairs of high quality DNA sequence were generated using Illumina technology to survey the poly (A)^+^ transcriptome of *P. bretschneideri*. A total of 90,227 unigenes were assembled. All known homologous genes in major metabolic pathways related to fruit development and maturation were identified. Furthermore, five DGE libraries were constructed and gene expression profiles at different fruit developmental stages were analyzed. These annotated transcriptome sequences and gene expression profiles provide useful information for identification of genes involved in quality trait development during fruit development and maturation in pear species.

## Methods

### Plant material and RNA extraction

**Figure 1:**
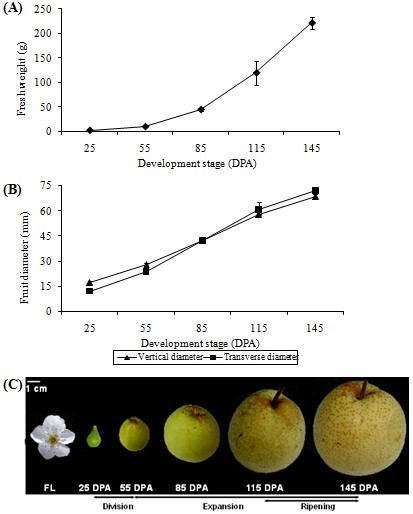
**Growth and development of ‘Dangshansuli’ pear fruit. (A)** Changes in fruit fresh weight. Mean of 5–10 fruits. Vertical bars represent standard deviations. **(B)** Changes in fruit transverse diameter and vertical diameter. Mean of 5–10 fruits. Vertical bars represent standard deviations. **(C)** Stages of fruit development from flower (FL) to ripening fruit and the corresponding days post anthesis (DPA) separated into 3 phases of development characterized by cell division, cell expansion, and fruit ripening.

All samples were collected from a 45-year old field grown *Pyrus bretschneideri* ‘Dangshansuli’ tree. Tissue samples of tender shoot, young leaf, expanded leaf, mature leaf, flower, and fruit were collected in 2010. Fruit samples were collected at 25, 55, 85, 115 and 145 DPA (days post anthesis) representing five developmental stages (Figure 1). All samples were immediately frozen in liquid nitrogen and kept at -80°C until use. Total RNA was extracted using modified CTAB method [32, 33]. RNA quality was monitored using the Agilent 2100 Bioanalyzer with a minimum integrity number value of 8.

### cDNA library construction, Illumina sequencing and *De novo* assembly

To obtain a complete gene expression profile, RNA from each tissue sample was pooled. The poly (A)^+^ RNA was isolated from 20 μg of the pooled RNA using Dynal oligo(dT) 25 beads (Invitrogen) according to the Illumina manufacturer’s instructions. The mRNA was then cleaved into short sequences using divalent cations at 70°C for 5 min. The cleaved RNA fragments were used for first strand cDNA synthesis using SuperScript III reverse transcriptase (Invitrogen) and N6 random hexamers (Takara). Second strand cDNA synthesis was performed using RNase H (Invitrogen) and DNA polymerase (Invitrogen). Subsequently, cDNA fragments were ligated to adapters after an end repair process. These products were purified and enriched using the QIAquick PCR Purification Kit to develop a cDNA library.

The cDNA library was sequenced from both 5′ and 3′ ends on the Illumina sequencing platform (HiSeq^TM^ 2000). Image deconvolution and Q-value (quality value assigned to each base) calculation were carried out in the Illumina data processing pipeline (version 1.6). Before assembly, adaptor sequences, empty reads, low quality sequences with N percentage (i.e., the percentage of nucleotides in read which could not be sequenced) over 5%, and those containing more than 20% bases with Q-value ≤ 10 were removed. *De novo* assembly of the cleaned reads was performed using SOAPdenovo software (version 1.03, http://soap.genomics.org.cn). The de Bruijn graph was firstly used to generate contigs [34]. The reads were then mapped back to the contigs, and the paired-end reads and contigs from the same transcript were further joined into scaffolds. The complete scaffolds were subsequently obtained after the paired-end reads were again used for gap fillings [34]. To reduce any sequence redundancy, the scaffolds were further assembled into unigenes using TGICL (copyright(c), http://www.tigr.org/tdb/tgi) [35]. Among them, the scaffolds with more than 70% similarity after multiple alignments were grouped into clusters, and others that cannot reach the threshold set and fall into any assembly should remain as a list of singletons. The assembled unigenes (larger than 200 bp) were deposited in the Transcriptome Shotgun Assembly Sequence Database (TSA) at NCBI with the accession number from JR595427 to JR673747.

### Functional annotation and classification

Unigenes that were larger than 150 bp were used for BLAST search and annotation against the NCBI nonredundant (nr) database with the BLASTX algorithm (http://www.ncbi.nlm.nih.gov/) using an E-value cut-off of 10^-5^. Functional annotation by gene ontology terms (GO; http://www.geneontology.org) was carried out based on the best hits from nr annotation using Blast2go software (http://www.blast2go.de/) [36], and the resultant GO id were further used for GO classification by WEGO [37] (http://wego.genomics.org.cn/cgi-bin/wego/index.pl). Annotation of COG and KEGG pathways was performed by sequence comparisons using BLASTX algorithm against Cluster of Orthologous Groups database and Kyoto Encyclopedia of Genes and Genomes database with an E-value threshold of 10^-5^. Annotations of all assembled sequences were deposited in the Transcriptome Shotgun Assembly Sequence Database (TSA) at NCBI and can be searched using the Gene-ID listed in Additional file 1.

### Digital gene expression (DGE) library construction and sequencing

Total RNA was extracted from fruit samples collected at five fruit developmental stages. The tag library was prepared using the Illumina gene expression sample prep kit. Briefly, the poly(A)^+^ RNA was purified from 6 μg of RNA using oligo(dT) magnetic beads. Double-stranded cDNA was directly synthesized on the beads and subsequently digested with NlaIII. All cDNA fragments with 3′ ends were purified with magnetic beads before their 5′ ends were ligated to Illumina adapter 1. A 21 bp tag with the adaptor 1 was produced after digested with MmeI (an enzyme that recognizes the junction of the Illumina adaptor 1 and the CATG site). The Illumina adapter 2 was then ligated to the 3′ ends of the tag. The library was amplified by PCR for 15 cycles and 90 bp strips were purified by PAGE gel electrophoresis. These strips were then digested and single-chain molecules were attached to the Illumina sequencing chip for sequencing. All raw tag data are available in Gene Expression Omnibus (GEO) at NCBI with the accession number: GSE33388.

### Analysis and mapping of DGE tags

To map DGE tags, sequencing-received raw image data were filtered to remove low quality tags (tags with unknown sequences ‘N’), empty tags (sequence with only the adaptors but no tags), and tags with only one copy number (probable sequencing error). For annotation, cleaned tags with CATG and the 21 bp tag sequence were mapped to our transcriptome reference database with no more than 1 nucleotide mismatch. All tags that mapped to reference sequences of multiple genes were filtered out and the remaining tags were designated as unambiguous tags for gene expression analysis. The number of unambiguous tags of each gene was calculated and then normalized to TPM (number of transcripts per million clean tags). The differentially expressed tags were used for mapping and annotation. A complete list of all differentially expressed genes is shown in Additional file 2, Additional file 3, Additional file 4 and Additional file 5.

### Evaluation of DGE libraries

To compare the gene expression in different fruit developmental stages, the frequency of each tag in different DGE libraries was statistically analyzed using the method of Audic *et al.*[38]. The false discovery rate (FDR) was used to determine the threshold *P*-value in multiple tests. The threshold determining the significance of differentially expressed genes was set at FDR ≤ 0.001 and log2 foldchange ≥ 2.

### Pathway enrichment analysis

Pathway enrichment analysis helps to identify significantly enriched metabolic pathways or signal transduction pathways in differentially expressed genes (DEGs) compared to the whole genome background. All DEGs mapped in the KEGG pathways with P-value ≤ 0.05 were considered statistically significantly enriched. The calculating formula is:

Here *N* is the number of all genes that with KEGG annotation, *n* is the number of DEGs in *N*, *M* is the number of all genes that were annotated to specific pathways, and *m* is number of DEGs in *M*.

## Results

### Illumina sequencing and *de novo* assembly

**Figure 2:**
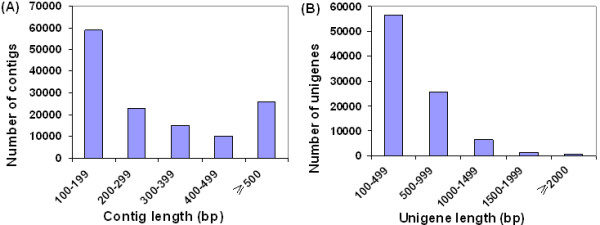
**Overview of the** ***P. bretschneideri*** **transcriptome sequencing and assembly. (A)** Size distribution of the contigs obtained from *de novo* assembly of high quality, clean reads. **(B)** Size distribution of the unigenes produced from further assembly of contigs by contig joining, gap filling, and scaffold clustering.

To obtain a global and comprehensive overview of the pear transcriptome, RNA was extracted from six different tissues including tender shoots, young leaves, expanded leaves, mature leaves, flowers, and fruits were equally mixed. A total of 60.8 millions of 90 bp raw reads were obtained from the Illumina paired-end sequencing with 5.47 gigabase pairs (Gbp) of raw data. After a stringent quality filtering process, 4.95 Gbp of clean data (90.5% of raw data) was remained. The Q20 percentage (an error probability lower than 1%) of the final sequence generated in this study (97.5%) was greater than those of tea (88%) [39], mouse (95%) [40], and Chinese bayberry (93%) [41], indicating that sequencing throughput and quality was acceptable for further analysis. *De novo* assembly of all clean reads by SOAPdenovo program produced 132,987 contigs (Table 1). The mean contig size was 338 bp and the N_50_ was 474 bp (i.e. 50% of the assembled bases were incorporated into contigs 474 bp or longer). Of all 132,987 contigs, 25,936 (19.5% of total) were larger than 500 bp (Figure 2A). After paired-end joining and gap-filling, the contigs were subsequently assembled into scaffolds. To reduce sequence redundancy, the assembled scaffolds were further clustered into 90,227 unigenes (larger than 150 bp) using TGICL software [35], including 17,619 clusters (mean size: 732 bp, N_50_: 902 bp) and 72,608 singletons (mean size: 452 bp, N_50_: 550 bp), as shown in Table 1. This unigene set had an average length of 508 bp and an N_50_ of 635 bp (Table 1). Of all 90,227 unigenes, 25,415 (28.2%) were ≥500 bp and 8,452 (9.4%) were ≥1,000 bp. The size distribution of these unigenes is shown in Figure 2B. To evaluate the quality of the dataset, the sequencing bias was analyzed by detecting random distribution of reads in assembled unigenes (Additional file 6). The 3′ and 5′ ends of all assembled unigenes contained relatively fewer reads and other positions showed a greater distribution, indicating that the assembled 90,227 unigenes show a great reliability and likely cover most of the transcriptome sequences.

**Table 1:** **Summary of sequence assembly of** ***P. bretschneideri*** **mRNA pooled from ten different tissues samples**

	Sequences (n)	Base pairs (bp)	Mean length (bp)	N50 (bp)
Raw sequencing reads	60,771,588	5,469,442,920	90	-
Clean reads	55,015,340	4,951,380,600	90	-
Contigs (≥100 bp)	132,987	44,990,137	338	474
Clusters (≥150 bp)	17,619	13,097,373	732	902
Singletons (≥150 bp)	72,608	32,694,777	452	550
Total unigenes (≥150 bp)	90,227	45,796,661	508	635

### Annotation of predicted proteins

Distinct gene sequences were first searched using BLASTx against the non-redundant (nr) NCBI nucleotide database with a cut-off E-value of 10^-5^. A total of 61,624 (68.3% of all distinct sequences) unigenes had a BLAST result above cut-off (Table 2, Additional file 1).

**Table 2:** **Summary of annotations of the** ***P. bretschneideri*** **unigenes**

	Sequences (n)	Annotations (n)	Functional classification
All assembled unigenes	90,227	-	-
Gene annotations against NR plant proteins	61,624	29,942	-
Gene annotations against NR Arabidopsis proteins	27,152	36,977	
Unique gene annotations against NR	61,636	61,636	
Gene annotations against UniProt	40,796	40,796	-
Gene annotations against InterPro	32,098	56,450	4,264 domains/families
Gene annotations against Pfam	30,985	37,194	3,406 domains/families
Gene annotations against COG	17,008	33,205	25 categories
Gene annotations against KEGG	31,215	31,215	121 pathways
GO annotations for NR protein hits	132,166	98,774	3 main categories, 44 sub-categories
All annotated Unigenes	62,077	-	-

**Figure 3:**
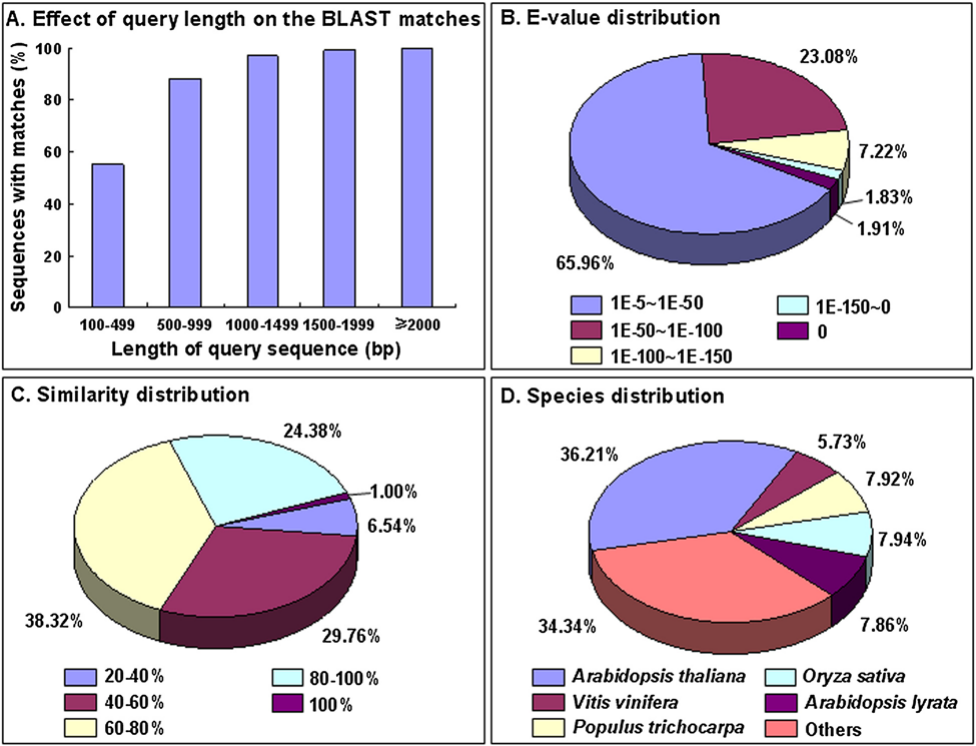
**Gene similarity of unigenes against the nr database. (A)** Effect of query sequence length on the percentage of significant matches. The cut-off value was set at 1.0E-5. The proportion of sequences with matches in the nr database at NCBI is greater among the assembled sequences with a greater length. **(B)** E-value distribution of the top BLAST hits for each unigene (E-value of 1.0E-5). **(C)** Similarity distribution of the best BLAST hits for each unigene. **(D)** Distribution of BLAST results by species is shown as the percentage of the total homologous sequences (with an E-value ≤1.0E-5). All plant proteins in the NCBI nr database were used for homology search and the best hit of each sequence was used for analysis.

Figure 3 indicates that the longer the assembled sequences, the greater the percentage of sequences with significant matches in the nr database. As shown in Figure 3A, only 54.9% of the unigenes shorter than 500 bp returned significant BLAST scores in the nr database. In contrast, the percentage of unigenes with significant BLAST scores increased sharply in which 88.3% for query sequences between 500 and 1,000 bp, 97.0% for query sequences between 1,000 and 1,500 bp, 99.2% for query sequences between 1,500 and 2,000 bp, and 100% for query sequences ≥2,000 bp. The E-value distribution of the top hits in the nr database showed that 34.0% of the mapped sequences have a great similarity (smaller than 1.0E-50) with 66.0% of the sequences ranged from 1.0E-5 to 1.0E-50 (Figure 3B). Furthermore, 25.4% of the query sequences have a similarity higher than 80%, while 74.6% of the hits have a similarity ranging from 20% to 80% (Figure 3C). Among species, 36.2% of pear distinct sequences have top matches (first hit) with sequences from *Arabidopsis thaliana*, while only 7.94%, 7.92%, 7.86%, and 5.73% of pear distinct sequences matched to those of *Oryza sativa*, *Populus trichocarpa*, *Arabidopsis lyrata*, and *Vitis vinifera*, respectively (Figure 3D).

### Conserved domain annotation

Conserved protein domains were identified in the *P. bretschneideri* unigenes against the InterPro, Pfam, and COGs databases. Searches against the InterPro database [42] revealed 32,098 top hits that were categorized into 4,264 domains/families (Table 2). Most domains contain 1–3 sequences, with a small proportion appearing more frequently. InterPro domains/families were ranked according to the number of unigenes included in each InterPro domain group. The 30 most abundant InterPro domains/families are provided in Table 3. Protein kinase and its subcategories Serine/threonine-protein kinase, Tyrosine-protein kinase, and Protein kinase-ATP binding site known to regulate the majority of cellular pathways were ranked in the top conserved domains. Cytochrome P450 and Myb-DNA-binding families that might contribute to extensive modifications of various secondary compounds and the “WD40-repeat” domain that is associated with the regulation of signal transduction, transcription, and proliferation [43] were also highly represented. By searching the Pfam database [44], 30,985 of the assembled unigenes matched entries that are corresponding to 3,406 different domains/families (Table 2).

**Table 3:** **Thirty most frequently occurring InterPro domains/families in the** ***P. bretschneideri*** **unigenes**

Conserved domain/family	Accession ID	Sequences (n)	Rank
Protein kinase, catalytic domain	IPR000719	1436	1
Serine/threonine-protein kinase-like domain	IPR017442	958	2
Leucine-rich repeat	IPR001611	890	3
Serine/threonine-protein kinase domain	IPR002290	699	4
Serine/threonine-protein kinase, active site	IPR008271	617	5
Pentatricopeptide repeat	IPR002885	590	6
Tyrosine-protein kinase, catalytic domain	IPR020635	589	7
Protein kinase, ATP binding site	IPR017441	515	8
Serine-threonine/tyrosine-protein kinase	IPR001245	510	9
RNA recognition motif domain	IPR000504	351	10
WD40 repeat, subgroup	IPR019781	348	11
Zinc finger, RING-type	IPR001841	344	12
Cytochrome P450	IPR001128	337	13
WD40-repeat-containing domain	IPR017986	332	14
NB-ARC	IPR002182	315	15
WD40 repeat 2	IPR019782	309	16
WD40 repeat	IPR001680	305	17
Zinc finger, C3HC4 RING-type	IPR018957	250	18
Myb, DNA-binding	IPR014778	207	19
Leucine-rich repeat-containing N-terminal, type 2	IPR013210	194	20
HTH transcriptional regulator, Myb-type, DNA-binding	IPR017930	187	21
SANT domain, DNA binding	IPR001005	181	22
EF-HAND 2	IPR018249	177	23
F-box domain, cyclin-like	IPR001810	165	24
Cytochrome P450, E-class, group I	IPR002401	163	25
EF-Hand 1, calcium-binding site	IPR018247	162	26
Tetratricopeptide repeat-containing	IPR013026	161	27
WD40 repeat, conserved site	IPR019775	156	28
Ankyrin repeat-containing domain	IPR020683	152	29
Helicase, superfamily 1/2, ATP-binding domain	IPR014021	148	30

### Gene ontology (GO), clusters of orthologous groups (COG) and Kyoto Encyclopedia of genes and genomes (KEGG) ontology (KO) classification

**Figure 4:**
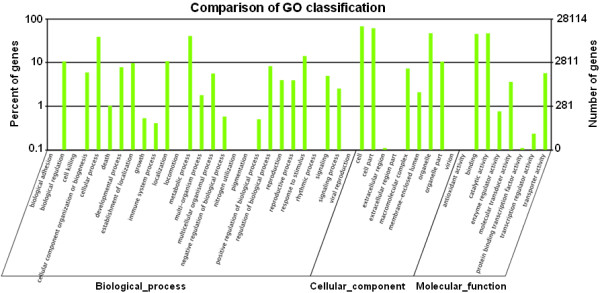
**Gene Ontology Classification of the** ***P. bretschneideri*** **transcriptome.** The results were summarized in three main GO categories (biological process, cellular component, molecular function) and 44 sub-categories. The right y-axis indicates the number of genes in a category. The left y-axis indicates the percentage of a specific category of genes in the main category.

Gene ontology (GO) assignments were used to classify functions of the predicted pear genes. Based on the sequence similarity, 28,114 sequences were categorized into 44 functional groups (Figure 4, Additional file 7) in three main categories (biological process, cellular component, and molecular function). Cellular process, metabolic process, cell, cell part, organelle, binding, and catalytic activity are most dominant terms presented in the three categories. Very few genes were clustered into “Biological adhesion”, “Cell killing”, “Locomoting”, “Nitrogen utilization”, “Pigmentation”, “Rhythmic process”, “Extracellular region part”, “Virion” or “Antioxidant activity”. It is also noticed that a high percentage of genes was clustered in the categories of “Biological regulation”, “Developmental process”, “Response to stimulus”, and “Transporter activity” (Figure 4, Additional file 7).

**Figure 5:**
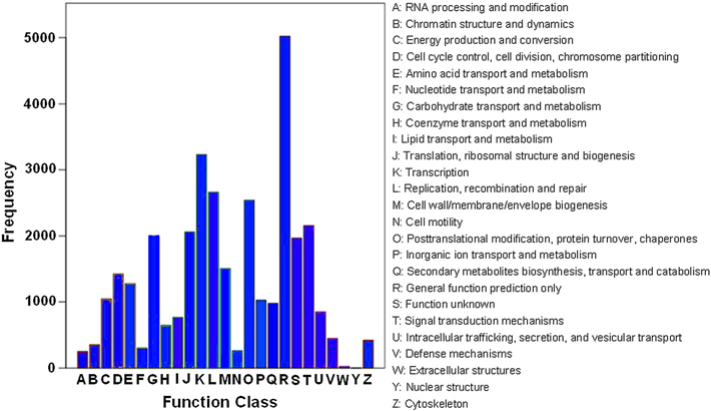
**COG Function Classification of the** ***P. bretschneideri*** **transcriptome.** A total of 33,205 unigenes showed significant homology (E-value ≤1.0E-5) to genes within one of the 25 categories (A-W, Y and Z) in the COGs database at NCBI.

To further evaluate the completeness of the pear transcriptome library and the effectiveness of the annotation process, a search was conducted to compare the annotated sequences against the COG database. A total of 33,205 annotated sequences were clustered into 25 COG categories (Figure 5) in which the cluster of “General function prediction” represented the largest group (5,021, 15.1%) followed by “Transcription” (3,230, 9.7%), “Replication, recombination and repair” (2,660, 8.0%), and “Posttranslational modification, protein turnover, and chaperones” (2,539, 7.7%). Nuclear structure (5, 0.015%), Extracellular structures (24, 0.072%), RNA processing and modification (251, 0.8%), and cell motility (263, 0.8%) appeared to be the smallest groups (Figure 5, Additional file 8).

A total of 90,227 annotated sequences were mapped to canonical pathways in Kyoto Encyclopedia of Genes and Genomes (KEGG) [45] to identify active pathways in white pear. Of which, 24,169 sequences were assigned to 121 KEGG pathways (Additional file 9). The most represented pathways by the unique sequences were metabolic pathways (5,488 members), biosynthesis of secondary metabolites (2,904 members), plant-pathogen interaction (2,389 members), and spliceosome (1,121 members). These annotations provide a valuable resource for investigating specific processes, functions, and pathways in pear growth and development.

### Analysis of metabolic pathway genes using *P. bretschneideri unigenes*

The overall quality of pear fruits is closely related to flavor, grit (stone cell) content, flesh texture, skin russet, and appearance [1, 46]. During fruit development and maturation, pear fruits undergo a series of physiological and biochemical changes including expansion of size, accumulation of soluble solids, change of pigments, and formation of aromatic volatiles [1, 2]. Most of these traits are inherited in a complex polygenic manner and controlled by multiple QTLs, which poses a significant challenge to traditional breeding [2, 47]. In order to better understand the genetic and molecular basis of these changes related to quality formation, a few pathways including several primary, intermediate, and secondary metabolic pathways that are related to pear quality development were analyzed. A total of 61,636 annotated unigenes were used to analyze metabolic pathway genes with simple keyword searches. Each search result was confirmed with a BLAST search.

**Figure 6:**
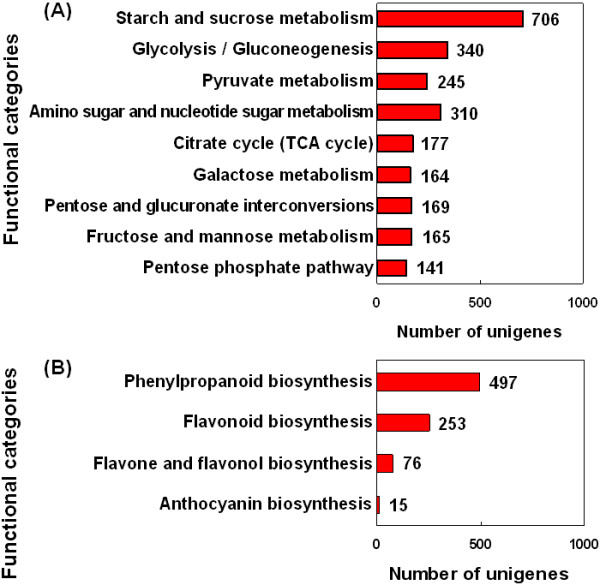
**Unigenes related to carbohydrate metabolism (A) and secondary metabolic pathways of flavonoids (B).**

Fruit development is involved in accumulation of starch and soluble sugar in fruits. Investigation of gene expression patterns in carbohydrate metabolisms will facilitate understanding of the fruit quality formation during the course of fruit development. According to KEGG, 2,417 unigenes were identified to be associated with carbohydrate metabolisms. These genes were classified into several metabolic pathways (Figure 6A). In this study, multiple unigenes involved in the main flavonoid biosynthesis pathways were annotated according to KEGG (Figure 6B). Flavonoids including phenylpropanoids, flavones, flavonols, and anthocyanins are important secondary metabolites that are directly involved in pear fruit quality development, such as grit (stone cell) content, skin russet, and appearance [1, 46]. Starch and sucrose metabolic pathways accounted for 29.2% of all 2,417 unigenes are another important group of carbohydrate metabolic pathways (Figure 6A). BLAST analysis showed that 18 key enzymes (outlined in Additional file 10) that are defined by 220 unigenes are involved in starch and sucrose metabolism pathways. Invertase (25), sucrose synthase (SS) (24), ADP glucose pyrophosphorylase (24), sucrose phosphate synthase (SPS) (23), and starch synthase (23) were ranked top five greatest numbers of singletons matching the gene description. Other primary metabolic pathways include glycolysis (150 genes), tricarboxylic acid cycle (89 genes), and pentose phosphate cycle (66 genes) pathways. The dataset also showed that the annotated sequences in the rest of three primary metabolic pathways have 0 to 25 singletons matching each gene (see Additional file 10). The shikimic acid pathway and the general phenylpropanoid pathway were the intermediate pathways of known topology involved in providing precursors for biosynthesis of flavonoids. A total of 114 unigenes were annotated in 16 enzymes that are involved in shikimic acid or aromatic amino acid pathway. Other 32 unigenes were involved in the general phenylpropanoid pathway and these unigenes might be involved in lignin biosynthesis (Additional file 10). The secondary metabolic pathways were selected, in which the simple six-carbon structure of hexoses were converted into a more complex 6:3:6 basic carbon structure of flavonoids. In the annotated *P. bretschneideri* transcriptome dataset, multiple unigenes encoding almost all known enzymes that are involved in main flavonoid biosynthesis pathways were identified (Additional file 10). All unigenes searched against the transcriptome database of *P. bretschneideri* will facilitate functional genomic studies and are particularly valuable for identifying genes or markers used for molecular breeding of pear species.

### Digital gene expression (DGE) library sequencing

**Figure 7:**
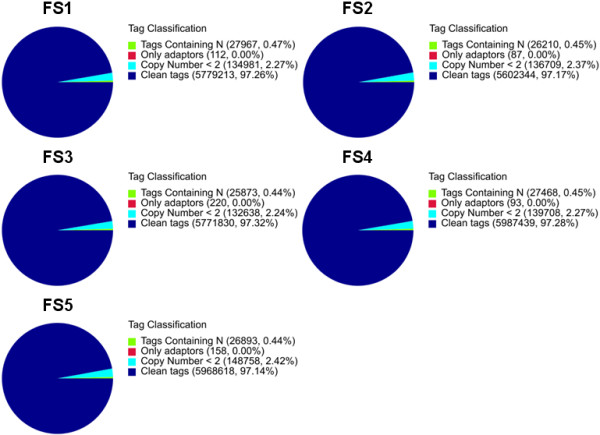
**Different components of the raw tags in each pear fruit sample.** Percentages of tags containing N (tags with unknown sequences), adaptors, a tag copy number <2, clean tags, and raw tags in each fruit stage library. The numbers in parentheses indicate the percentage of each type of tag among the total raw tags.

Digital gene expression (DGE) method generates direct gene expression measurements, which avoids the inherent limitation of microarray analysis. Five DGE libraries corresponding to five developmental stages of pear fruits were sequenced with 5.7 to 6.2 million raw tags per library (Table 4). Five stages and their DGE accession numbers deposited in GEO were: fruit stage 1 (GSM825779), fruit stage 2 (GSM825780), fruit stage 3 (GSM825781), fruit stage 4 (GSM825782), and fruit stage 5 (GSM825783). The number of clean tags per library ranged from 5.6 to 6.0 million after filtering out the low quality reads (Figure 7). Of all clean tags, 4.5 to 4.9 million tags were mapped to unigenes (Table 4). The total number of tags with unique nucleotide sequences ranged from 122,997 to 130,176 (Table 4).

**Table 4:** **Statistics of** ***P. bretschneideri*** **DGE sequencing**

Summary	FS1	FS2	FS3	FS4	FS5
Total number of raw tags	5,942,273	5,765,350	5,930,561	6,154,708	6,144,427
Total number of distinct raw tags	275,061	279,671	273,457	278,272	284,436
Total number of clean tags	5,779,213	5,602,344	5,771,830	5,987,439	5,968,618
Total number of distinct clean tags	127,209	130,176	128,372	125,656	122,997
Clean tag/Raw tag	97.26%	96.40%	97.32%	97.28%	97.14%
Total number of tags mapping to genes	4,601,197	4,514,247	4,742,242	4,916,898	4,676,676
% of tags mapping to genes	79.62	80.58	82.18	82.12	78.35
Total number of distinct tags mapping to gene	73,993	76,128	77,039	74,448	68,916
% of distinct tags mapping to gene	58.17	58.48	60.01	59.25	56.03
Number of all tag-mapped genes	34,781	35,530	36,335	35,415	32,705
% of all tag-mapped genes	38.55	39.38	40.27	39.25	36.25
Number of unambiguous tags mapping to gene	3,875,820	3,766,999	3,956,567	4,137,841	3,856,928
% of unambiguous tags mapping to gene	67.06	67.24	68.55	69,11	64.62
Number of distinct unambiguous tags mapping to gene	61,872	63,617	64,242	61,873	56,894
% of distinct unambiguous tags mapping to gene	48.64	48.87	50.04	49.24	46.26
Number of unambiguous tag-mapped genes	26,836	27,541	28,175	27,331	24,975
% of unambiguous tag-mapped genes	29.74	30.52	31.23	30.29	27.68
Number of unknown tags	1,178,016	1,088,097	1,028,588	1,070,541	1,291,942
% of unknown tags	20.38	19.42	17.82	17.88	21.65
Number of distinct unknown tags	53,216	54,048	51,333	51,208	54,081
% of distinct unknown tags	41.83	41.52	39.99	40.75	43.97

Notes: Statistics of raw tags, clean tags, tags mapped to unigenes, unambiguous tags and unknown tags. Abbreviations: DGE, digital gene expression; FS1, fruit stage 1; FS2, fruit stage 2; FS3, fruit stage 3; FS4, fruit stage 4; FS5, fruit stage 5.

**Figure 8:**
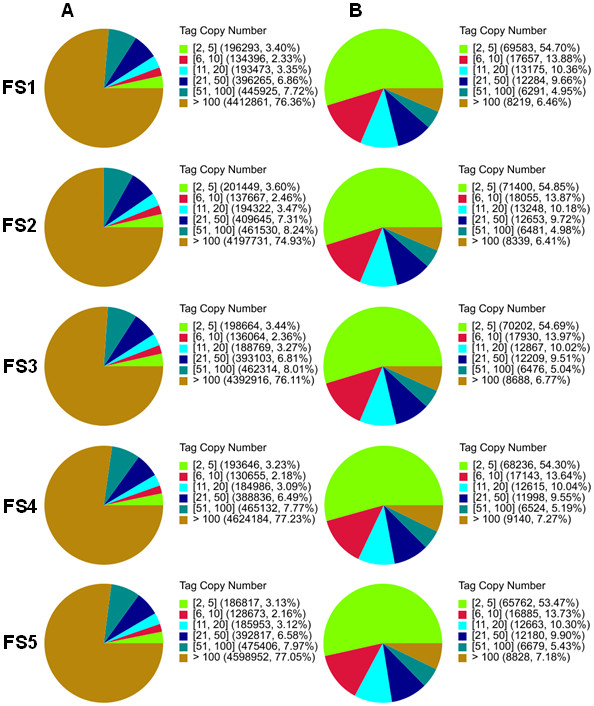
**Distribution of total clean tags and distinct clean tags over different tag abundance groups. (A)** Distribution of total tags. Numbers in the square brackets indicate the range of copy number in that group. For example, [2,5; green] means all the tags in this group have 2 to 5 copies. Numbers in the parentheses show the total number of tags that fall into this copy number range and the percentage of the library represented. **(B)** Distribution of distinct tags. Numbers in the square brackets indicate the range of copy number in this category. Numbers in the parentheses show the total types of tags in that category and the percentage of the library encompassed.

During gene expression, heterogeneity and redundancy of mRNA are two significant characteristics with the majority of mRNA being expressed at a low level. To evaluate the normality of the DGE data, distribution of the clean tag expression was evaluated (Figure 8). All five DGE libraries showed a similar pattern in distribution of total tags or distinct tags (2–5 copies, 6–10 copies, etc., as indicated by different colors). The highly expressed tags (copy number >100; Figure 8A) represented greater than 74.9% of the clean tags in each library; however, the number of high copy number clean tags that appeared in one developmental stage (hence, distinct Figure 8B) did not exceed 7.3%. In contrast, tags with a low copy number (<5; Figure 8A) represented the majority of distinct clean tags in each library (Figure 8B).

Tag sequences in the five DGE libraries were mapped to the transcriptome reference database generated in the above-mentioned Illumina sequencing. The reference database contains 162,456 distinct sequences with 142,331 unambiguous reference tags. Among the distinct tags (68,916 to 77,039) generated from the Illumina sequencing in the five libraries of fruit developmental stages, 32,705 to 36,335 distinct tags were mapped to individual genes in the reference database (Table 4). Tags mapped to a unique sequence are the most critical subset of the DGE libraries as they can explicitly identify a transcript. Up to 31.2% (28,175) of the sequences in the transcriptome reference tag database could be unequivocally identified by a unique tag (Table 4).

**Figure 9:**
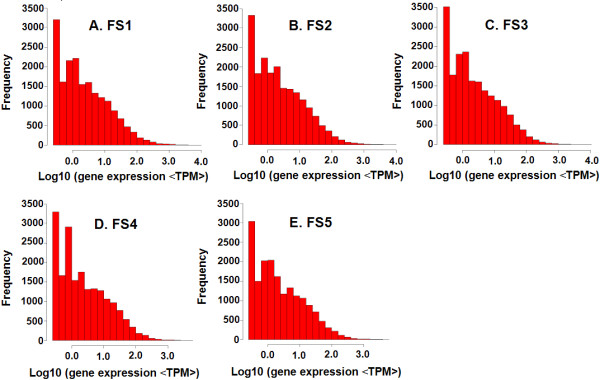
**The level of expression for each gene.** Gene expression level was determined by calculating the number of unambiguous tags for each gene and then normalized to TPM (transcript copies per million tags). FS1: fruit stage 1, FS2: fruit stage 2, FS3: fruit stage 3, FS4: fruit stage 4, and FS5: fruit stage 5.

To confirm a proportional increase of the number of detected genes to an increase of total tag number, a saturation analysis was performed. Additional file 11 shows a trend of saturation where the increase of the number of detected genes stopped when the number of reads reached 4 million. The gene expression level was determined by calculating the number of unambiguous tags of each gene and then normalizing the expression level to the number of transcripts per million tags (TPM). As summarized in Figure 9, the majority of genes (those toward the right of each graph) resulted in fewer than ten copies and only a small proportion of genes are highly expressed.

### Gene expression profile changes between developmental stages

To profile the gene expression pattern during different developmental stages of pear fruit, the number of clean tags for each gene was calculated and its differentially expressed tags between two samples of two adjacent stages were identified using an algorithm developed by Audic *et al.*[38].

**Figure 10:**
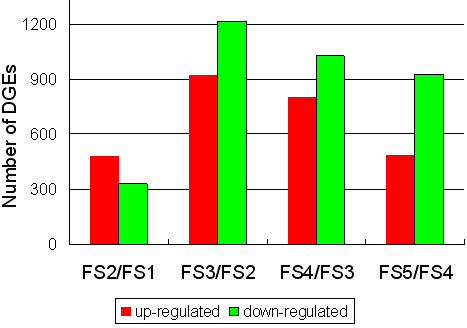
**Changes in gene expression profile among the different developmental stages.** Unigenes up-regulated (red) and down-regulated (down) between two adjacent fruit developmental stages were quantified. DGEs: digital gene expressions, FS1: fruit stage 1, FS2: fruit stage 2, FS3: fruit stage 3, FS4: fruit stage 4, and FS5: fruit stage 5.

A total of 2,980 tags were detected to be significantly changed between fruit stage 2 (FS2) and fruit stage 1 (FS1) libraries. Those tags were mapped to 810 genes with 478 genes being up-regulated and 332 genes being down-regulated (Figure 10, Additional file 2). Five of the ten most up-regulated genes have defined functions: a dehydration-induced RD22-like protein, a metallothionein-like protein type 3, a nonspecific lipid-transfer protein precursor, a cytochrome P450, and a Cytochrome P450 82A4. Only one of the ten most down-regulated genes has defined functions: a zinc finger protein. In addition, a total of 14 genes among the 20 differentially expressed genes had unknown functions or no annotations (Additional file 12). According to the GO classification, most of the genes are correlated to cellular components: cytoplasm, plastid, organelle, or intracellular organelle. In the KO classification, 119 gene sets were significantly enriched and most of these genes were correlated to metabolic processes, i.e., metabolic pathways, photosynthesis-antenna proteins, and photosynthesis (Additional file 13).

The comparative analysis between fruit stage 3 (FS3) and FS2 libraries revealed that 2,135 genes showed significant expression changes. Among these genes, 923 and 1,212 genes were up-regulated and down-regulated, respectively, in FS3 compared to FS2 (Figure 10, Additional file 3). Among the ten most up-regulated and ten most down-regulated genes, seven annotated genes were up-regulated in FS3: two prunin 2 precursor [*Prunus dulcis*], one unnamed protein product [*Vitis vinifera*], one dehydration-responsive protein RD22 [*Prunus persica*], one mitochondrial carrier protein [*Ricinus communis*], one conserved hypothetical protein [*Ricinus communis*], and one tonoplast intrinsic protein (putative) [*Ricinus communis*]. Seven functionally defined genes were down-regulated in FS3, including a flavonoid 3-hydroxylase gene [*Ricinus communis*], a RNA binding protein (putative) [*Ricinus communis*], an APETALA2 (AP2) domain class transcription factor [*Malus* x *domestica*], a CBF/DREB1 transcription factor [*Malus baccata*], an anthocyanidin synthase gene [*Pyrus communis*], an Auxin-binding protein ABP19a, and an NADH dehydrogenase subunit 6 [*Nicotiana tabacum*]. Six genes were also blasted without annotation (Additional file 12). Based on the GO functional classification, almost all genes were involved in cellular components, i.e., extracellular region, membrane part, anchored to membrane and external encapsulating structure. Among genes enriched in KO, significant changes were observed in genes of metabolic pathways, such as biosynthesis of phenylpropanoids, biosynthesis of plant hormones, flavonoid biosynthesis, and starch and sucrose metabolism (Additional file 14).

Between the FS4 and FS3 libraries, 1,824 genes demonstrated significant changes. In the FS4 library, 797 and 1,027 genes were up-regulated and down-regulated, respectively, in comparison with the FS3 library (Figure 10, Additional file 4). Nine genes among the ten most differentially up- and down-regulated genes showed no similarity (Additional file 12). Among the up-regulated genes, five were hypothetical protein from *Vitis vinifera* and *Populus trichocarpa*, in which only one matched a flavonoid 3-hydroxylase gene from *Ricinus communis*, two down-regulated genes were predicted to encode proteins found in *Ricinus communis* and *Populus trichocarpa*, an unnamed protein product [*Vitis vinifera*], a laccase 1a [*Populus trichocarpa*] and an Nonspecific lipid-transfer protein precursor [*Ricinus communis*] (Additional file 12). Almost all gene sets enriched in GO were correlated to plastid, plastid part, thylakoid, and chloroplast, while the gene sets enriched in KO were related to metabolic pathways (Additional file 15).

The comparison between the FS5 and FS4 libraries also revealed significant variations in gene expression. A total of 1,411 genes, including 484 up-regulated and 927 down-regulated, were identified in FS5 compared to FS4 (Figure 10, Additional file 5). Eight genes among the ten most differentially up- and down-regulated genes showed no similarity (Additional file 12). Among the ten up-regulated genes, one aligned with a beta-D-xylosidase gene [*Pyrus pyrifolia*], one with an AP2 domain class transcription factor [*Malus* x *domestica*], and two genes were unnamed protein product [*Vitis vinifera*] and conserved hypothetical protein [*Ricinus communis*]. Among the ten down-regulated genes, three aligned with dehydration-responsive protein RD22, two with prunin precursors, one with a delta-12 oleate desaturase [*Gossypium hirsutum*], and two were unnamed protein product or hypothetical protein. Most genes enriched in GO were related (or functional in) chloroplast stroma and photosystem. Genes in enriched KO showing significant changes between the FS5 and FS4 libraries were related to cysteine and methionine metabolism, photosynthesis-antenna proteins and isoquinoline alkaloid biosynthesis (Additional file 16).

### Expression profiling during ripening

In this study, differences in the number and expression profile of DEGs were observed at five developmental stages of pear fruit. 28,743 unigenes were expressed during fruit ripening, with 810, 2,135, 1,824, and 1,411 showing differential expression between 25 and 55 DPA, 55 and 85 DPA, 85 and 115 DPA, and 115 and 145 DPA, respectively. It showed that fewer genes expressed in the cell division stage (25 to 55 DPA) than in the period of cell expansion (55 to 145 DPA) and fruit ripening (115 to 145 DPA) (Figures 1 and 10).

To characterize the levels of gene expression, the number of unambiguous tags of each gene was calculated and normalized to the number of TPM. Based on this analysis, the gene expression levels in the five fruit developmental stages were categorized to rare (TPM < 5), low (TPM > 5–50), moderate (TPM > 50–100), and high (TPM >100) (Additional file 17). The largest portion of transcripts (20,431 out of 28,744, 71.08%) exhibited rare expression, and followed by low expression (6,483 out of 28,744, 22.55%). Only a small fraction of transcripts was expressed at moderate (3.21%) and high (3.15%) levels.

For a global view of expression patterns, 4 groups were defined according to their expression profiles and some of representative genes were selected and shown in Table 5. Genes in Group I are up-regulated during the fruit development period, which suggests that a few metabolic processes are enhanced and catalytic activity increases. Some genes involved in the process of fruit maturation have this kind of expression patterns, such as SPS, starch synthase, alkaline invertase (Alnv), phosphofructokinase, maltose transporter that are related to starch and sucrose metabolism, 4-hydroxyphenylpyruvate dioxygenase, leucoanthocyanidin dioxygenase (anthocyanin and lignin metabolism), expansin (cell wall loosening protein), and ACC oxidase (ethylene biosynthesis). Genes in Group II are down-regulated, such as genes involved in auxin transport (auxin influx transport protein), sugar metabolism (SS, pyrophosphate-dependent phosphofructo-1-kinase), volatile biosynthesis (alcohol acyl-transferase), and phenylpropanoid pathway (arogenate/prephenate dehydratase, UDPG flavonoid 7-*O*-glucosyltransferase). Genes in Group III showed a high level of gene expression in the middle period of fruit development. These genes are involved in sugar metabolism (NAD-dependent sorbitol dehydrogenase), phytohormone metabolism (DELLA protein, gibberellin oxidase), stress resistance (salt-tolerance protein, polyphenol oxidase), and flavonoid metabolism (*p*-hydroxyphenylpyruvate dioxygenase, cytochrome P450). Genes in Group IV constitutively expressed through the entire fruit developmental period, such as genes that participate in basal metabolism and membrane stability showed this expression pattern. The information on the gene expression level and pattern during the entire fruit development period will help further elucidate gene functions and well understand the molecular mechanisms of fruit development and maturation in pear species.

**Table 5:** **Selected genes with different expression trends in five fruit developmental stages**

Gene ID	Gene annotation	TPM (transcript per million clean tag)				
		FS1	FS2	FS3	FS4	FS5
	***Up-regulated pattern***					
Unigene86203_PB_AHAU	Sucrose phosphate synthase	0.35	0.54	1.39	1.84	0.84
Unigene51453_PB_AHAU	Starch synthase	6.75	11.07	21.31	9.69	30.83
Unigene28216_PB_AHAU	Alkaline invertase	66.27	107.1	149.35	209.27	190.33
Unigene31510_PB_AHAU	Phosphofructokinase	1.21	0.89	15.25	21.55	25.8
Unigene79028_PB_AHAU	Maltose transporter	30.11	34.45	35.17	72.65	123.31
Unigene7706_PB_AHAU	Aldehyde dehydrogenase	48.28	59.44	87.67	93.03	102.54
Unigene11393_PB_AHAU	Expansin	21.63	20.35	39.33	74.99	148.11
Unigene84426_PB_AHAU	ACC oxidase	8.82	13.57	13.69	6.68	159
Unigene41650_PB_AHAU	4-Hydroxyphenylpyruvate dioxygenase	0	0.54	3.47	4.01	5.36
Unigene49425_PB_AHAU	Leucoanthocyanidin dioxygenase	1.73	11.07	13.34	49.77	36.19
	***Down-regulated pattern***					
Unigene78545_PB_AHAU	Auxin influx transport protein	130.12	120.31	136.7	65.97	32.84
Unigene28595_PB_AHAU	Sucrose synthase	1120.57	775.21	201.84	18.2	5.7
Unigene2239_PB_AHAU	Pyrophosphate-dependent phosphofructo-1-kinase	28.38	16.42	2.08	3.01	1.01
Unigene82187_PB_AHAU	Alcohol acyl-transferase	179.78	215.27	80.22	12.86	19.43
Unigene21328_PB_AHAU	Alcohol acyl-transferase	185.49	154.04	102.91	14.53	9.55
Unigene41185_PB_AHAU	Alcohol acyl-transferase	958.95	738.98	286.74	201.42	84.78
Unigene24664_PB_AHAU	Flavonoid 3-hydroxylase	114.55	62.47	0	3.67	0.34
Unigene89425_PB_AHAU	COL domain class transcription factor	100.88	80.86	13.34	10.52	4.36
Unigene40707_PB_AHAU	Arogenate/prephenate dehydratase	309.04	207.23	45.74	17.04	9.21
Unigene42300_PB_AHAU	UDPG flavonoid 7-*O*-glucosyltransferase	125.62	192.42	72.25	34.07	67.52
	***Low-high-low pattern***					
Unigene28017_PB_AHAU	NAD-dependent sorbitol dehydrogenase	9.52	6.96	21.66	11.69	4.02
Unigene48458_PB_AHAU	NAD-dependent sorbitol dehydrogenase	65.58	60.15	208.43	94.87	28.15
Unigene40996_PB_AHAU	*p*-Hydroxyphenylpyruvate dioxygenase	0.52	1.25	12.13	6.68	8.88
Unigene36705_PB_AHAU	Cytochrome P450	36.68	39.98	208.77	167.35	69.7
Unigene1335_PB_AHAU	Cytochrome P450	12.46	8.21	77.96	21.38	11.9
Unigene15706_PB_AHAU	Salt-tolerance protein	14.36	16.78	83.86	18.2	13.57
Unigene6651_PB_AHAU	DELLA protein	44.99	40.7	80.74	32.4	51.44
Unigene29424_PB_AHAU	Polyphenol oxidase	33.57	28.02	79.52	40.92	14.07
Unigene2416_PB_AHAU	Gibberellin oxidase	0.35	1.61	77.96	17.7	0
	***Constitutive pattern***					
Unigene1535_PB_AHAU	S6 ribosomal protein	650.26	506.57	717.1	432.91	403.28
Unigene42402_PB_AHAU	Structural molecule activity	505.09	381.09	387.23	437.58	380.99
Unigene41042_PB_AHAU	DEAD-box RNA helicase-like protein	481.38	399.83	458.09	641.68	533.46
Unigene41056_PB_AHAU	Large ribosomal subunit	333.09	256.14	291.59	243.18	225.51
Unigene40598_PB_AHAU	Plasma membrane ATPase	29.93	22.13	20.62	50.1	13.57
Unigene41711_PB_AHAU	Plasma membrane intrinsic protein	19.38	28.92	41.23	23.88	21.11
Unigene8634_PB_AHAU	Plasma intrinsic protein	1121.95	866.6	964.51	974.21	799.18
Unigene13819_PB_AHAU	BHLH domain class transcription factor	212.49	243.11	169.79	168.85	206.92
Unigene3327_PB_AHAU	60S ribosomal protein L35a	180.65	182.6	166.5	154.99	138.73
Unigene40848_PB_AHAU	Ribosomal protein S21e	173.03	218.12	188.33	170.36	182.62

Notes: Gene ID is the accession number of each assembled unigenes deposited in the Transcriptome Shotgun Assembly Sequence Database (TSA) at NCBI.

Abbreviations: DGE, digital gene expression; FS1, fruit stage 1; FS2, fruit stage 2; FS3, fruit stage 3; FS4, fruit stage 4; FS5, fruit stage 5.

## Discussion

### Illumina sequencing and function annotation

In this study, one ‘Dangshansuli’ pear cDNA library and five DGE libraries (from fruit samples collected at 25, 55, 85, 115 and 145 DPA) were constructed using RNA-Seq technology. These constructed libraries were subjected to comparative gene expression studies. As a result, we obtained 90,227 unigenes including 17,619 clusters and 72,608 singletons. 62,077 out of 90,227 assembled unigenes were annotated. Of which, 61,624 were annotated to the non-redundant (nr) NCBI database and 28,114, 33,205, and 24,196 were annotated to GO, COG and KEGG databases, respectively. For the remaining 28,150 unigenes (31.2% of all 90,227 assembled unigenes), the absence of significant homology to existing genes could be caused by several factors. Obviously, the length and completeness of the assembled unigenes were one main factor, which can be seen from the increasing proportion of sequences with matches in the NR database as the length of uingenes increased (Figure 3A). However, the assembled unigenes shorter than 500 bp added up to 64,812 (71.8% of all 90,227 assembled unigenes) (Figure 2B), and some of them were too short to allow statistically meaningful matches. In addition, for some unigenes, lack of homologous sequences in the public databases may indicate specific roles for them in *P. bretschneideri*.

Unigenes identified through conserved domain annotation showed that most abundant InterPro domains/families were Protein kinase and its subcategories that are known to regulate the majority of cellular pathways. Cytochrome P450, Myb-DNA-binding families (MYB), and “WD40-repeat” domains were also very abundant. MYB and WD40-repeat proteins contributed to the extensive modification and regulation of various secondary compounds in many pathways have been validated to be key roles in anthocyanin biosynthesis by regulating anthocyanin structural genes [48]. MYB proteins also appear to be versatile in regulating secondary metabolism, cellular morphogenesis, meristem formation, and the cell cycle [49]. Recent research showed that Cytochrome P450 encoded enzymes that are responsible for the conversion of the mogroside backbone to various mogrosides [26].

ESTs sequences had been intensively used for transcriptional analysis, candidate gene discovery, and gene functional analysis. However, since RNA-Seq technology was developed, only 4,413 expressed sequence tags (ESTs) of *Pyrus* plants have been deposited in GenBank due to the fact that EST analyses can only identify limited number of candidate genes that are involved in complex biosynthetic pathways. Using RNA-seq, over one billion nucleotides of high-quality DNA sequence per analysis/experiment can be generated [8], which has dramatically improved the efficiency of gene discovery and functional analysis [9, 10]. Furthermore, RNA-Seq was less expensive, more efficient, and allowed faster gene discovery than traditional EST analysis. RNA-seq recently has been widely used for transcriptome profiling analysis in many plant species [15, 26, 41, 48–52].

### Genes related to metabolic pathway

In the present study, a total of 31,215 unigenes were assigned to 121 KEGG pathways, among which 2,417 unigenes were identified to be associated with carbohydrate metabolisms. A total of 34.8% and 29.2% of all 2,417 unigenes are involved in flavonoid biosynthesis and starch and sucrose metabolism, respectively. Invertase (25 unigenes), SS (24 unigenes), ADP glucose pyrophosphorylase (24 unigenes), SPS (23 unigenes), and starch synthase (23 unigenes) were ranked top five enzymes in 18 key enzymes (see Additional file 10) involved in starch and sucrose metabolism pathways.

Sugar is one of the most important biochemical components that determine fruit quality. A series of enzymes control sucrose metabolism during fruit development and maturation. Invertase, SS, and SPS are three key enzymes that are deeply involved in fruit sucrose matabolism. For example, invertase (*β*-D-fructofuranosidase) cleaves sucrose to glucose and fructose irreversible, while soluble acid invertase (AIV) presumably hydrolyzes sucrose to hexose for cell growth and development [53, 54]. A decrease of soluble AIV activity was correlated with a rapid increase of sucrose during fruit maturation in Japanese pear [55, 56]. SS is involved in the movement of sucrose to diverse pathways important for metabolic structure and storage functions in plant cells [57]. It has been reported that SS plays a role in sucrose cleavage rather than sucrose synthesis [58, 59]; however, one report suggested that SS was also involved in sucrose synthesis in mature peach fruit where a high level of sucrose was accumulated [60]. In pear fruit, SS also appears to be involved in sucrose synthesis since SS activity increased along with a sucrose accumulation in pear fruit [60]. Recent research in potato also showed that SS strongly determines the intracellular levels of UDP-glucose, ADP-glucose, and starch, and total yield in potato tubers [61]. SPS also plays a major role in sucrose biosynthesis. Both SPS and SS are two important determinants of sucrose accumulation in Asian pear fruit. In 23 pear cultivars, the activity of SS was closely correlated with sucrose content, while SPS showed a weak correlation [60].

In the family Rosaceae, sorbitol is a major carbohydrate of translocated photosynthates. Sorbitol is converted into other sugars in the fruit by sorbitol-metabolizing enzymes, in which sorbitol oxidase and NAD-dependent sorbitol dehydrogenase are two major players [62, 63]. Research also revealed that three gene families, sorbitol transport (SOT), sorbitol dehydrogenase (SDH), and sorbitol-6-phosphate dehydrogenase (S6PDH), showed more impacts on sugar metabolism in pear than in non-rosaceous species [64]. Wu *et al.* (2013) [64] identified four *S6PDH* genes through genome sequencing; however, no unigenes coding *S6PDH* were detected in this study. This may be caused by the difference in gene assembly or annotation. Yamaki and Moriguchi (1989) reported that NAD-dependent sorbitol dehydrogenase that converts sorbitol to fructose showed a high activity throughout the entire fruit developing period in Japanese pear fruit. However, the activity of sorbitol oxidase activity that is about one-tenth of NAD-dependent sorbitol dehydrogenase, was high in immature fruit, but decreased during the fruit expansion period and increased again during the fruit maturation stage [63]. In peach fruit, sorbitol oxidase activity was relatively high, but other sorbitol-related enzymes were barely detectable [65].

In this study, two important enzymes, ADP glucose pyrophosphorylase (24 unigenes) and starch synthase (23 unigenes) that are involved in starch metabolic pathways were identified. In plants, regulation of starch metabolism is complex. In synthesis of starch, ADP-glucose was the glucosyl donor for the elongation of α-1, 4-glucosidic chains [66]. The first committed step is ADP-glucose synthesis that is catalyzed by ADP-glucose pyrophosphorylase (ADPGlc PPase) [67]. As a glucose donor, ADPG molecule is transferred to non-reducing end of the (1–4) glucan primer by starch synthase catalyses, thus formation of a long-chain amylase. Ghosh and Preiss (1966) showed that the reaction catalyzed by ADPGlc PPase is a regulation step in starch synthesis in higher plants as well as in the cyanobacteria [68–70]. Most of the plant ADPGlc PPases are allosterically regulated by 3-PGA and inorganic orthophosphate (Pi) [69, 70]. Some reports also suggested that in higher plants the enzyme activity can also be regulated by its reductive state [71–73].

In this study, multiple unigenes in the main flavonoid biosynthesis pathways were annotated according to KEGG (Figure 6B). Flavonoids including phenylpropanoids, flavones, flavonols, and anthocyanins are important secondary metabolites that are directly involved in the development of fruit quality, such as color, flavor, and health beneficial ingredients [1, 46, 74]. These compounds are also involved in the formation of undesirable brown pigments in fruits following bruising or cutting and/or storage [75]. Information on phenylpropanoid metabolism in pear is limited. Nishitani *et al.* (2010) have studied the importance of phenylpropanoid metabolism in pear fruit ripening using oligoarray analysis [75]. In present study, a large number (497) of unigenes involved in phenylpropanoid biosynthesis were detected. Lignin biosynthesis in the phenylpropanoid pathway is one of the important factors for pear fruit quality. Lignin is the primary component of stone cells in pear fruit [76] and its synthesis has direct influence on formation and content of stone cells, ultimately influencing pear fruit quality [64]. Anthocyanin biosynthesis is essential for fruit coloration. Red coloration is determined by the content and composition of anthocyanins in the fruit skin [77]. Most enzymes in the anthocyanin biosynthetic pathway in pears are well studied [76]. For example, Feng *et al.* (2010) reported that anthocyanin biosynthesis in pears is regulated by a R2R3-MYB transcription factor *Py*MYB10 [78].

### Digital gene expression (DGE) at different stages of pear fruits

Analyses of KEGG pathways showed that DEGs were observed in several different pathways including some metabolic pathways, such as biosynthesis of phenylpropanoids, plant hormones, and flavonoids; starch and sucrose metabolism; cysteine and methionine metabolism; biosynthesis of photosynthesis-antenna proteins and isoquinoline alkaloid. These are closely related to fruit development in pear and other species [60, 61, 66, 74, 79].

Comparative analysis showed that dehydration-responsive protein RD22 was significantly up-regulated in pear fruit between FS3 and FS2 and significantly down-regulated between the FS3 and FS2 libraries. This showed that this gene involved in the whole expansion stage of pear fruit, so we infer that it may play important roles in the process of pear fruit growth and development. Although some research reported that dehydration-responsive protein RD22 was related to stress/defense response [80–82]. The role of this gene in pear fruit development remains unknown. AP2 was well known for its roles in floral organ identity and develop. In this study, we found that AP2 domain class transcription factor was significantly down-regulated and up-regulated in the fruit developmental stage of FS3/FS2 and FS5/FS4, respectively, which indicated that the AP2 domain class transcription factor was involved in fruit growth and development in pear. Recent research showed that AP2 was involved in many aspects of fruit development in other species. For example, Chung *et al.* (2010) proved that a tomato (*Solanum lycopersicum*) APETALA2/ERF gene (SlAP2a) is a negative regulator of fruit ripening [83]. AP2 genes are also involved in seed mass and yield development *via* regulation of embryo cell number and cell size [84]. Rohrmann *et al.* (2011) reported that some genes in the AP2-EREBP family responsive to ethylene also showed the altered expression from the green fruit developmental stage onwards [85]. Ripoll *et al.* (2011) found that AP2 acts to prevent overgrowth of replum by negatively regulating BP and RPL, two genes that normally act to promote replum formation in *Arabidopsis*. AP2 also acts to prevent overgrowth of the valve margin by repressing the expression of the valve margin identity gene [86]. These studies indicated that AP2 domain class transcription factor is an important regulatory factor in fruit development. Other genes identified in this study, such as metallothionein-like protein type 3, nonspecific lipid-transfer protein precursor, cytochrome P450, zinc finger protein, prunin 2 precursor, flavonoid 3-hydroxylase gene, CBF/DREB1 transcription factor, anthocyanidin synthase gene, etc. may have their own roles in the process of pear fruit growth and development. These roles need further verification.

## Conclusions

In this study, the transcriptome profile of Chinese white pear (*P. bretschneideri*) was investigated using Solexa/Illumina RNA-seq and DGE deep sequencing technologies. A total of 90,227 unigenes were assembled and 62,077 unigenes were annotated. The results demonstrated the feasibility of using Illumina sequencing-based DGE system for gene expression profiling and provided new directions for functional analysis of genes involved in pear fruit development. These findings provide a substantial contribution to existing sequence resources of pear species and will certainly valuable for elucidation of molecular mechanisms in fruit development and maturation in pear or related species. Therefore, this study clearly evidenced that Illumina sequencing technology could be applied as a rapid and cost-effective method for *de novo* transcriptome analysis of non-model plant species that lack of prior genome annotation.

## Availability of supporting data

The assembled unigenes (larger than 200 bp) were deposited in the Transcriptome Shotgun Assembly Sequence Database (TSA) at NCBI, with accession numbers from JR595427 to JR673747.

## Electronic supplementary material

Additional file 1: Top BLAST hits from NCBI nr database. BLAST results against the NCBI nr database for all distinct sequences with a cut-off E value above 10^-5^. (XLS 17 MB)

Additional file 2: Differentially expressed genes between fruit stage 2 (FS2) and fruit stage 1 (FS1). TPM: transcript copies per million tags. Raw intensity: the total number of tags sequenced for each gene. FDR: false discovery rate. FDR < 0.001 and the absolute value of log2Ratio ≤1 were used as the threshold to judge the significance of gene expression difference. In order to calculate the log2Ratio and FDR, TPM value of 0.001 instead of 0 for genes that do not express in one sample was used. (XLS 6 MB)

Additional file 3: Differentially expressed genes between fruit stage 3 (FS3) and fruit stage 2 (FS2). TPM: transcript copies per million tags. Raw intensity: the total number of tags sequenced for each gene. FDR: false discovery rate. FDR < 0.001 and the absolute value of log2Ratio ≤1 were used as the threshold to judge the significance of gene expression difference. In order to calculate the log2Ratio and FDR, TPM value of 0.001 instead of 0 for genes that do not express in one sample was used. (XLS 7 MB)

Additional file 4: Differentially expressed genes between fruit stage 4 (FS4) and fruit stage 3 (FS3). TPM: transcript copies per million tags. Raw intensity: the total number of tags sequenced for each gene. FDR: false discovery rate. FDR < 0.001 and the absolute value of log2Ratio ≤1 were used as the threshold to judge the significance of gene expression difference. In order to calculate the log2Ratio and FDR, TPM value of 0.001 instead of 0 for genes that do not express in one sample was used. (XLS 7 MB)

Additional file 5: Differentially expressed genes between fruit stage 5 (FS5) and fruit stage 4 (FS4). TPM: transcript copies per million tags. Raw intensity: the total number of tags sequenced for each gene. FDR: false discovery rate. FDR <0.001 and the absolute value of log2Ratio ≤1 were used as the threshold to judge the significance of gene expression difference. In order to calculate the log2Ratio and FDR, TPM value of 0.001 instead of 0 for genes that do not express in one sample was used. (XLS 6 MB)

Additional file 6: Random distribution of Illumina sequencing reads in the assembled unigenes. The *x*-axis indicates the relative position of sequencing reads in the assembled unigenes. The orientation of unigene is from 5′ to 3′ end. (DOC 38 KB)

Additional file 7: GO annotation of unigenes. Combination of the GO annotation provided by Blast2Go and InterProScan. (XLS 3 MB)

Additional file 8: COG Functional classification of the *P. bretschneideri* transcriptome. A total of 33,205 unigenes showed significant similarity to one of the 25 COG categories at NCBI (E-value ≤1.0e^-5^). (XLS 251 KB)

Additional file 9: Kyoto Encyclopedia of Genes and Genomes (KEGG) ontology (KO) classification of the *P. bretschneideri* transcriptome. A total of 24,169 sequences were assigned to 121 KEGG pathways. (XLS 806 KB)

Additional file 10: Putative genes coding for structural enzymes in key pathways of primary, intermediate, and secondary metabolism in the *P. bretschneideri* datasets. (DOC 105 KB)

Additional file 11: Correlation between the number of detected genes and sequencing volume (total tag number). All figures show a trend of saturation. Once sequencing reaches 4 million reads, the number of detected genes almost ceases to increase. (DOC 210 KB)

Additional file 12: Top ten differentially expressed genes in each two-way library comparison. (DOC 174 KB)

Additional file 13: Gene set enrichment analysis comparing FS2 and FS1. (XLS 20 KB)

Additional file 14: Gene set enrichment analysis comparing FS3 and FS2. (XLS 22 KB)

Additional file 15: Gene set enrichment analysis comparing FS4 and FS3. (XLS 22 KB)

Additional file 16: Gene set enrichment analysis comparing FS5 and FS4. (XLS 18 KB)

Additional file 17: Differentially expressed genes of five fruit developmental stages. TPM: transcript copies per million tags. (XLS 8 MB)
