# Supplementary material for: Transcriptome profiling of fruit development and maturation in Chinese white pear (Pyrus bretschneideri Rehd)
Source: BMC Genomics. 2013 Nov 23;14(1):823. doi: 10.1186/1471-2164-14-823 (PMC4046828; doi:10.1186/1471-2164-14-823)
Supplement: Supplementary file 6 — Additional file 6: Random distribution of Illumina sequencing reads in the assembled unigenes. The x-axis indicates the relative position of sequencing reads in the assembled unigenes. The orientation of unigene is from 5′ to 3′ end. (DOC 38 KB) [file 12864_2013_5518_MOESM6_ESM.doc]

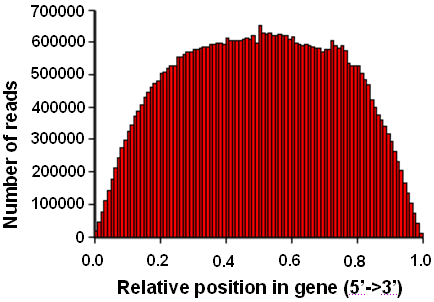


**Additional file 1. Random distribution of Illumina sequencing reads in the assembled unigenes.** The *x*-axis indicates the relative position of sequencing reads in the assembled unigenes. The orientation of unigene is from 5’ end to 3’ end.
