# Supplementary material for: Transcriptome profiling of fruit development and maturation in Chinese white pear (Pyrus bretschneideri Rehd)
Source: BMC Genomics. 2013 Nov 23;14(1):823. doi: 10.1186/1471-2164-14-823 (PMC4046828; doi:10.1186/1471-2164-14-823)
Supplement: Supplementary file 10 — Additional file 10: Putative genes coding for structural enzymes in key pathways of primary, intermediate, and secondary metabolism in the P. bretschneideri datasets. (DOC 105 KB) [file 12864_2013_5518_MOESM10_ESM.doc]

Additional file 11. Putative genes coding for structural enzymes in key pathways of primary, intermediate, and secondary metabolism in the *P. bretschneideri* datasets.

| **Pathways: Structural Enzymes** | **E.C.** | **Total Unigenes ^a^** |
| --- | --- | --- |
| Starch and sucrose metabolism |  |  |
| Fructose 1, 6-bisphosphate aldolase | 4.1.2.13 | 15 |
| Phosphohexose isomerase | 5.3.1.9 | 8 |
| Phosphoglucomutase | 5.4.2.2 | 5 |
| UDP-glucose pyrophosphorylase | 2.7.7.9 | 6 |
| Sucrose phosphate synthase | 2.4.1.14 | 23 |
| Sucrose synthase | 2.4.1.13 | 24 |
| Sucrose phosphatase | 3.1.3.24 | 0 |
| Sucrose phosphorylase | 2.4.1.7 | 0 |
| ADP glucose pyrophosphorylase | 2.7.7.27 | 24 |
| Starch synthase | [2.4.1.21](http://enzyme.expasy.org/EC/2.4.1.21) | 23 |
| Glycogen branching enzyme | 2.4.1.25 | 11 |
| Invertase | 3.2.1.26 | 25 |
| Sorbitol-6-phosphate dehydrogenase | 1.1.1.140 | 0 |
| NAD-dependent sorbitol dehydrogenase | [1.1.1.14](http://www.genome.jp/dbget-bin/www_bget?ec:1.1.1.14) | 21 |
| Sorbitol oxidase | 1.1.3.- | 9 |
| Hexokinase | 2.7.1.1 | 13 |
| Fructokinase | 2.7.1.4 | 13 |
| Glucokinase | 2.7.1.2 | 0 |
| Glycolysis (EMP pathway) |  |  |
| Hexokinase | 2.7.1.1 | 13 |
| Glucose-6-phosphoisomerase | 5.3.1.9 | 8 |
| 6-Phosphofructokinase | 2.7.1.11 | 25 |
| Fructose 1, 6-biphosphate aldolase | 4.1.2.13 | 15 |
| Triose phosphate isomerase | 5.3.1.1 | 8 |
| Glyceraldehyde 3-P dehydrogenase | 1.2.1.12 | 15 |
| Phosphoglycerate kinase | 2.7.2.3 | 9 |
| Phosphoglycerate mutase | 5.4.2.1 | 22 |
| Enolase | 4.2.1.11 | 14 |
| Pyruvate kinase | 2.7.1.40 | 21 |
| Tricarboxylic acid cycle (Krebs cycle) |  |  |
| Citrate synthase | 2.3.3.1 | 11 |
| Aconitase | 4.2.1.3 | 12 |
| Isocitrate dehydrogenase | 1.1.1.42 | 18 |
| α-Ketoglutarate dehydrogenase | 1.2.4.2 | 13 |
| Succinyl-CoA synthetase | 6.2.1.5 | 8 |
| Succinate dehydrogenase | 1.3.5.1 | 7 |
| Fumarase | 4.2.1.2 | 3 |
| Malate dehydrogenase | 1.1.1.37 | 17 |
| Oxidative/ nonoxidative pentose phosphate pathway |  |  |
| Glucose 6-P-1-dehydrogenase | 1.1.1.49 | 21 |
| 6-Phosphogluconolactonase | 3.1.1.31 | 8 |
| 6-Phosphogluconate dehydrogenase | 1.1.1.44 | 7 |
| Ribose-5-P isomerase | 5.3.1.6 | 12 |
| Ribose-5-P 3-epimerase | 5.1.3.1 | 9 |
| Transketolase | 2.2.1.1 | 4 |
| Transaldolase | 2.2.1.2 | 5 |
| Shikimic acid pathway: aromatic amino acid biosynthesis |  |  |
| 3-Deoxy-D-arabino-heptulosonate 7-P synthase | 2.5.1.54 | 15 |
| 3-Dehydroquinate synthase | 4.2.3.4 | 3 |
| 3-Dehydroquinate dehydratase | 4.2.1.10 | 18 |
| Shikimate 5 dehydrogenase | 1.1.1.25 | 16 |
| Shikimate kinase | 2.7.1.71 | 7 |
| 5-Enolpyruvoylshikimate 3-P synthase | 2.5.1.19 | 7 |
| Chorismate synthase | 4.2.3.5 | 6 |
| Chorismate mutase | 5.4.99.5 | 9 |
| Prephenate dehydratase | 4.2.1.51 | 0 |
| Prephenate dehydrogenase | 1.3.1.12 | 0 |
| Aromatic amino acid transaminase | 2.6.1.57 | 0 |
| Anthranilate synthase | 4.1.3.27 | 14 |
| Anthranilate phosphoribosyl transferase | 2.4.2.18 | 4 |
| Phosphoribosylanthranilate synthase | 5.3.1.24 | 1 |
| Indol-3-glycerol phosphate synthase | 4.1.1.48 | 4 |
| Tryptophan synthase | 4.2.1.20 | 10 |
| General phenylpropanoid pathway |  |  |
| Phenylalanine ammonia-lyase | 4.3.1.24 | 8 |
| Cinnamate 4-hydroxylase (NADPH cytochrome P450 reductase) | 1.14.13.11 | 5 |
| 4-Coumarate coenzyme A ligase | 6.2.1.12 | 19 |
| General phenylpropanoid pathway: lignin biosynthesis |  |  |
| Caffeic acid *O*-methyltransferase | 2.1.1.68 | 17 |
| Caffeoyl-CoA *O*-methyltransferase | 2.1.1.104 | 11 |
| Cinnamoyl-CoA reductase | 1.2.1.44 | 38 |
| Ferulate 5-hydroxylase | 1.14.-.- | 16 |
| 5-Hydroxy feruloyl CoA *O*-methyltransferase | No E.C. | 0 |
| Cinnamyl alcohol dehydrogenase | 1.1.1.195 | 36 |
| 5-Hydroxy coniferyl alcohol *O*-methyltransferase | No E.C. | 0 |
| 5-Hydroxy coniferaldehyde *O*-methyltransferase | No E.C. | 0 |
| Flavonoid biosynthetic pathway: flavonol biosynthesis |  |  |
| Naringenin chalcone synthase | 2.3.1.74 | 23 |
| Chalcone isomerase | 5.5.1.6 | 4 |
| Flavanone 3-hydroxylase | 1.14.11.9 | 13 |
| Flavonol 3-hydroxylase | 1.14.13.21 | 43 |
| Flavonol synthase | 1.14.11.23 | 16 |
| Flavonol 3-*O*-glucosyltransferase | 2.4.1.91 | 9 |
| Flavonoid biosynthetic pathway: anthocyanin biosynthesis |  |  |
| Dihydroflavonol 4-reductase | 1.1.1.219 | 23 |
| Leucoanthocyanidin dioxygenase | 1.14.11.19 | 17 |
| UDP-flavonol 3-*O*-glucosyltransferase | 2.4.1.91 | 9 |
| Anthocyanin 5-*O*-glucosyltransferase | 2.4.1.- | 8 |
| Anthocyanin 5-aromatic acyltransferase | 2.3.1.153 | 0 |
| Anthocyanin permease | No E.C. | 0 |
| Flavonoid biosynthetic pathway: proanthocyanidin biosynthesis |  |  |
| Anthocyanidin reductase | 1.3.1.77 | 9 |
| Leucoanthocyanindin reductase | 1.17.1.3 | 23 |

**^a^** Represents the total number of putative unigenes based on BLASTX definition selecting only E values of E < 1.0E-5.
