# Supplementary material for: Transcriptome profiling of fruit development and maturation in Chinese white pear (Pyrus bretschneideri Rehd)
Source: BMC Genomics. 2013 Nov 23;14(1):823. doi: 10.1186/1471-2164-14-823 (PMC4046828; doi:10.1186/1471-2164-14-823)
Supplement: Supplementary file 11 — Additional file 11: Correlation between the number of detected genes and sequencing volume (total tag number). All figures show a trend of saturation. Once sequencing reaches 4 million reads, the number of detected genes almost ceases to increase. (DOC 210 KB) [file 12864_2013_5518_MOESM11_ESM.doc]

**FS1**

**FS2**

**FS3**

**FS4**

**FS5**

**Additional file 6. Relationship between the number of detected genes and sequencing volume (total tag number).** All figures show a trend of saturation. Once sequencing reaches 4 million reads, the number of detected genes almost ceases to increase. Abbreviations: FS1, fruit stage 1; FS2, fruit stage 2; FS3, fruit stage 3; FS4, fruit stage 4; FS5, fruit stage 5.
