# Supplementary material for: Transcriptome profiling of fruit development and maturation in Chinese white pear (Pyrus bretschneideri Rehd)
Source: BMC Genomics. 2013 Nov 23;14(1):823. doi: 10.1186/1471-2164-14-823 (PMC4046828; doi:10.1186/1471-2164-14-823)
Supplement: Supplementary file 12 — Additional file 12: Top ten differentially expressed genes in each two-way library comparison. (DOC 174 KB) [file 12864_2013_5518_MOESM12_ESM.doc]

**Additional file 12. Top ten differentially expressed genes in each library of comparisons.**

| **Comparison** | **Unigene**  **ID** | **Hit Number** | **Discription** | **FDR** | **Fold Change** |
| --- | --- | --- | --- | --- | --- |
| **FS2 /FS1** | **Unigene68613^a^** | **-** | **-** | **0** | **10.978** |
|  | **Unigene21058^a^** | **gi\|18447761\|gb\|AAL67991.1\|** | **dehydration-induced protein RD22-like protein [*Gossypium hirsutum*]** | **0** | **10.387** |
|  | **Unigene25564^a^** | **gi\|225429860\|ref\|XP_002283317.1\|** | **PREDICTED: hypothetical protein [*Vitis vinifera*]** | **0** | **10.267** |
|  | **Unigene28772^a^** | **gi\|3334262\|sp\|O24059.1\|** | **Metallothionein-like protein type 3** | **0** | **10.267** |
|  | **Unigene42372^a^** | **-** | **-** | **0** | **10.267** |
|  | **Unigene74838^a^** | **gi\|255567206\|ref\|XP_002524584.1\|** | **Nonspecific lipid-transfer protein precursor, putative [*Ricinus communis*]** | **0** | **10.136** |
|  | **Unigene50420^a^** | **gi\|225434490\|ref\|XP_002275261.1\|** | **PREDICTED: hypothetical protein isoform 1 [*Vitis vinifera*]** | **0** | **9.858** |
|  | **Unigene48317^a^** | **gi\|118488559\|gb\|ABK96092.1\|** | **unknown [*Populus trichocarpa*]** | **8.34E-13** | **9.649** |
|  | **Unigene65461^a^** | **gi\|255582040\|ref\|XP_002531817.1\|** | **cytochrome P450, putative [*Ricinus communis*]** | **2.35E-11** | **9.480** |
|  | **Unigene41389^a^** | **gi\|5915845\|sp\|O49859.1\|** | **Cytochrome P450 82A4** | **1.30E-09** | **9.246** |
|  | **Unigene61317^b^** | **-** | **-** | **1.01E-09** | **-9.28309** |
|  | **Unigene34629^b^** | **gi\|225424504\|ref\|XP_002285219.1\|** | **PREDICTED: hypothetical protein [*Vitis vinifera*]** | **1.35E-08** | **-9.11374** |
|  | **Unigene62123^b^** | **gi\|255562023\|ref\|XP_002522020.1\|** | **zinc finger protein, putative [*Ricinus communis*]** | **3.37E-07** | **-8.86728** |
|  | **Unigene35454^b^** | **gi\|297743998\|emb\|CBI36968.3\|** | **unnamed protein product [*Vitis vinifera*]** | **1.22E-06** | **-8.75822** |
|  | **Unigene52263^b^** | **-** | **-** | **2.31E-06** | **-8.69697** |
|  | **Unigene42565^b^** | **gi\|296086467\|emb\|CBI32056.3\|** | **unnamed protein product [*Vitis vinifera*]** | **5.41E-05** | **-8.36194** |
|  | **Unigene59782^b^** | **-** | **-** | **5.41E-05** | **-8.36194** |
|  | **Unigene41607^b^** | **gi\|217071218\|gb\|ACJ83969.1\|** | **unknown [*Medicago truncatula*]** | **5.40E-05** | **-8.36194** |
|  | **Unigene7362^b^** | **gi\|224064358\|ref\|XP_002301436.1\|** | **predicted protein [*Populus trichocarpa*]** | **5.40E-05** | **-8.36194** |
|  | **Unigene14250^b^** | **gi\|224127864\|ref\|XP_002329196.1\|** | **predicted protein [*Populus trichocarpa*]** | **1.01E-04** | **-8.28077** |
| **FS3 /FS2** | **Unigene14622^a^** | **gi|307159114|gb|ADN39441.1|** | **prunin 2 precursor [*Prunus dulcis*]** | **3.62E-14** | **16.12816** |
|  | **Unigene40701^a^** | **gi\|307159112\|gb\|ADN39440.1\|** | **prunin 1 precursor [*Prunus dulcis*]** | **3.60E-14** | **15.17418** |
|  | **Unigene4446^a^** | **-** | **-** | **3.56E-14** | **14.81528** |
|  | **Unigene89212^a^** | **gi\|296084806\|emb\|CBI27688.3\|** | **unnamed protein product [*Vitis vinifera*]** | **3.61E-14** | **14.0396** |
|  | **Unigene85601^a^** | **gi\|16588826\|gb\|AAL26909.1\|** | **dehydration-responsive protein RD22 [*Prunus persica*]** | **3.52E-14** | **11.88303** |
|  | **Unigene54387^a^** | **gi\|255550607\|ref\|XP_002516353.1\|** | **mitochondrial carrier protein, putative [*Ricinus communis*]** | **3.59E-14** | **11.82217** |
|  | **Unigene21383^a^** | **gi\|255555176\|ref\|XP_002518625.1\|** | **conserved hypothetical protein [*Ricinus communis*]** | **3.56E-14** | **11.79442** |
|  | **Unigene85136^a^** | **-** | **-** | **3.55E-14** | **11.78013** |
|  | **Unigene1746^a^** | **gi\|255569391\|ref\|XP_002525663.1\|** | **tonoplast intrinsic protein, putative [*Ricinus communis*]** | **3.58E-14** | **11.63072** |
|  | **Unigene78632^a^** | **-** | **-** | **3.53E-14** | **11.61471** |
|  | **Unigene24664^b^** | **gi\|255549934\|ref\|XP_002516018.1\|** | **flavonoid 3-hydroxylase, putative [*Ricinus communis*]** | **1.97E-106** | **-12.6089** |
|  | **Unigene35528^b^** | **gi\|297744219\|emb\|CBI37189.3\|** | **unnamed protein product [*Vitis vinifera*]** | **7.33E-86** | **-12.3024** |
|  | **Unigene73648^b^** | **-** | **-** | **7.60E-67** | **-11.9458** |
|  | **Unigene2312^b^** | **gi\|255552015\|ref\|XP_002517052.1\|** | **RNA binding protein, putative [*Ricinus communis*]** | **6.42E-51** | **-11.5589** |
|  | **Unigene53142^b^** | **-** | **-** | **2.03E-30** | **-10.8305** |
|  | **Unigene14611^b^** | **gi\|292668891\|gb\|ADE41100.1\|** | **AP2 domain class transcription factor [*Malus* x *domestica*]** | **3.35E-29** | **-10.7723** |
|  | **Unigene61139^b^** | **gi\|148357329\|gb\|ABQ59086.1\|** | **CBF/DREB1 transcription factor [*Malus baccata*]** | **6.27E-25** | **-10.5498** |
|  | **Unigene50089^b^** | **gi\|81295652\|gb\|ABB70119.1\|** | **anthocyanidin synthase [*Pyrus communis*]** | **8.47E-23** | **-10.4242** |
|  | **Unigene22608^b^** | **gi\|18203684\|sp\|Q9ZRA4.1\|** | **Auxin-binding protein ABP19a** | **1.70E-22** | **-10.4062** |
|  | **Unigene65501^b^** | **gi\|57014000\|ref\|YP_173471.1\|** | **NADH dehydrogenase subunit 6 [*Nicotiana tabacum*]** | **5.67E-21** | **-10.3072** |
| **FS4 /FS3** | **Unigene78925^a^** | **gi|225451015|ref|XP_002280966.1|** | **PREDICTED: hypothetical protein [*Vitis vinifera*]** | **0** | **14.35425** |
|  | **Unigene9633^a^** | **gi\|296089951\|emb\|CBI39770.3\|** | **unnamed protein product [*Vitis vinifera*]** | **2.58E-11** | **9.41996** |
|  | **Unigene77342^a^** | **-** | **-** | **1.76E-10** | **9.310613** |
|  | **Unigene44201^a^** | **-** | **-** | **1.10E-07** | **8.870365** |
|  | **Unigene25278^a^** | **gi\|224067842\|ref\|XP_002302560.1\|** | **predicted protein [*Populus trichocarpa*]** | **2.10E-07** | **8.816984** |
|  | **Unigene53142^a^** | **-** | **-** | **2.71E-06** | **8.584963** |
|  | **Unigene29830^a^** | **gi\|147821544\|emb\|CAN72253.1\|** | **hypothetical protein VITISV_034184 [*Vitis vinifera*]** | **2.71E-06** | **8.584963** |
|  | **Unigene64840^a^** | **-** | **-** | **5.11E-06** | **8.519636** |
|  | **Unigene24664^a^** | **gi\|255549934\|ref\|XP_002516018.1\|** | **flavonoid 3-hydroxylase, putative [*Ricinus communis*]** | **5.10E-06** | **8.519636** |
|  | **Unigene40766^a^** | **gi\|225426255\|ref\|XP_002264299.1\|** | **PREDICTED: hypothetical protein [*Vitis vinifera*]** | **9.60E-06** | **8.455327** |
|  | **Unigene74838^b^** | **gi\|255567206\|ref\|XP_002524584.1\|** | **Nonspecific lipid-transfer protein precursor, putative [*Ricinus communis*]** | **3.53E-104** | **-12.5285** |
|  | **Unigene60571^b^** | **-** | **-** | **1.63E-87** | **-12.2796** |
|  | **Unigene81803^b^** | **gi\|225466807\|ref\|XP_002273111.1\|** | **PREDICTED: hypothetical protein [*Vitis vinifera*]** | **5.01E-77** | **-12.0977** |
|  | **Unigene63858^b^** | **-** | **-** | **6.28E-70** | **-11.9604** |
|  | **Unigene87981^b^** | **gi\|297746143\|emb\|CBI16199.3\|** | **unnamed protein product [*Vitis vinifera*]** | **1.90E-63** | **-11.8222** |
|  | **Unigene81388^b^** | **gi\|224124110\|ref\|XP_002330107.1\|** | **predicted protein [*Populus trichocarpa*]** | **3.34E-58** | **-11.7** |
|  | **Unigene65555^b^** | **-** | **-** | **1.42E-49** | **-11.4722** |
|  | **Unigene41249^b^** | **-** | **-** | **9.88E-40** | **-11.1592** |
|  | **Unigene12830^b^** | **-** | **-** | **6.90E-38** | **-11.0921** |
|  | **Unigene82491^b^** | **gi\|224139024\|ref\|XP_002322961.1\|** | **laccase 1a [*Populus trichocarpa*]** | **4.04E-35** | **-10.9851** |
| **FS5 /FS4** | **Unigene43652^a^** | **-** | **-** | **1.52E-13** | **9.651052** |
|  | **Unigene88574^a^** | **-** | **-** | **1.00E-10** | **9.31515** |
|  | **Unigene40865^a^** | **-** | **-** | **3.89E-08** | **8.924813** |
|  | **Unigene42665^a^** | **gi\|157041199\|dbj\|BAF79669.1\|** | **beta-D-xylosidase [*Pyrus pyrifolia*]** | **3.74E-06** | **8.527477** |
|  | **Unigene88434^a^** | **-** | **-** | **1.35E-05** | **8.388017** |
|  | **Unigene66634^a^** | **gi\|297742227\|emb\|CBI34376.3\|** | **unnamed protein product [*Vitis vinifera*]** | **2.57E-05** | **8.312883** |
|  | **Unigene41923^a^** | **-** | **-** | **0.000172** | **8.066089** |
|  | **Unigene25203^a^** | **gi\|292668911\|gb\|ADE41110.1\|** | **AP2 domain class transcription factor [*Malus* x *domestica*]** | **0.000172** | **8.066089** |
|  | **Unigene4680^a^** | **gi\|255627007\|gb\|ACU13848.1\|** | **unknown [*Glycine max*]** | **0.000172** | **8.066089** |
|  | **Unigene40021^a^** | **gi\|255603438\|ref\|XP_002538048.1\|** | **conserved hypothetical protein [*Ricinus communis*]** | **0.000172** | **8.066089** |
|  | **Unigene28533^b^** | **gi\|16588826\|gb\|AAL26909.1\|** | **dehydration-responsive protein RD22 [*Prunus persica*]** | **0** | **-16.7215** |
|  | **Unigene14622^b^** | **gi\|307159114\|gb\|ADN39441.1\|** | **prunin 2 precursor [*Prunus dulcis*]** | **0** | **-16.2387** |
|  | **Unigene21058^b^** | **gi\|18447761\|gb\|AAL67991.1\|** | **dehydration-induced protein RD22-like protein [*Gossypium hirsutum*]** | **0** | **-14.6222** |
|  | **Unigene89212^b^** | **gi\|296084806\|emb\|CBI27688.3\|** | **unnamed protein product [*Vitis vinifera*]** | **0** | **-14.5183** |
|  | **Unigene78925^b^** | **gi\|225451015\|ref\|XP_002280966.1\|** | **PREDICTED: hypothetical protein [*Vitis vinifera*]** | **0** | **-14.3542** |
|  | **Unigene4446^b^** | **-** | **-** | **0** | **-14.087** |
|  | **Unigene40701^b^** | **gi\|307159112\|gb\|ADN39440.1\|** | **prunin 1 precursor [*Prunus dulcis*]** | **4.78E-188** | **-13.3635** |
|  | **Unigene48317^b^** | **gi\|118488559\|gb\|ABK96092.1\|** | **unknown [*Populus trichocarpa*]** | **6.01E-186** | **-13.3473** |
|  | **Unigene27584^b^** | **gi\|310657311\|gb\|ADP02395.1\|** | **delta-12 oleate desaturase [*Gossypium hirsutum*]** | **1.72E-126** | **-12.7966** |
|  | **Unigene85601^b^** | **gi\|16588826\|gb\|AAL26909.1\|** | **dehydration-responsive protein RD22 [*Prunus persica*]** | **6.88E-117** | **-12.6839** |

**The corner marks of gene ID: ‘a’ represents up-regulated genes and ‘b’ represents down-regulated genes. The expression fold changes were performed with log 2 ratio.**
